# Investigating the Potential Mechanism of Oxymatrine in Alleviating Heat Stress Injury Based on Network Pharmacology, Molecular Docking, and In Vitro Validation

**DOI:** 10.3390/ijms27135919

**Published:** 2026-06-30

**Authors:** Sheng Cheng, Xingxing Song, Wenying Qiu, Xiaowan Liu, Guangneng Peng, Jialiang Xin

**Affiliations:** Key Laboratory of Animal Disease and Human Health of Sichuan Province, College of Veterinary Medicine, Sichuan Agricultural University, Chengdu 611130, China; chengsheng@stu.sicau.edu.cn (S.C.); sxx96721@163.com (X.S.); wingsqiu@163.com (W.Q.); guoguowan1990@163.com (X.L.)

**Keywords:** heat stress, oxymatrine, network pharmacology, molecular docking, PI3K-AKT signaling pathway, ferroptosis

## Abstract

Global warming has increasingly positioned heat stress (HS) as a major threat to public health, as it can inflict damage on multiple organs including the kidneys, liver, and heart. However, effective targeted therapeutic strategies remain limited. This investigation employed an integrated approach combining Network pharmacology, in silico binding simulations, and cell-based assays to elucidate the cytoprotective properties and molecular basis of oxymatrine action under heat-stressed conditions. Network analysis identified 36 overlapping targets common to oxymatrine and the pathological processes of HS-related acute kidney injury (AKI), acute liver injury (ALI), and acute myocardial injury (AMI). These targets were strongly enriched in the PI3K-AKT signaling cascade. Molecular docking showed that oxymatrine binds tightly to key pathway proteins such as PIK3CA and GSK3B, with Vina scores below −8 kcal/mol. In 293T cells, the half-maximal cytotoxic concentration (CC_50_) of oxymatrine exceeded 2000 μM. Under heat stress, oxymatrine (31.25–1000 μM) dose-dependently increased cell viability by about 30% and significantly lowered *HSP90* and *HSP70* expression. Similar protective effects were observed in H9C2 cardiomyocytes under heat stress. RT-qPCR further confirmed that oxymatrine reduced the transcript levels of PI3K-AKT pathway-related genes, including *CASP3*, *EGFR*, *RXRα*, and *MMP9* in 293T cells. We also found 18 overlapping targets between oxymatrine and ferroptosis, most of which matched the core targets above. Molecular docking analysis predicted binding of oxymatrine to the ferroptosis regulator GPX4. Together, these results suggested that oxymatrine potentially alleviates HS injury by modulating the PI3K-AKT signaling pathway andregulating potential ferroptotic targets such as GPX4.

## 1. Introduction

Heat stress (HS) is a systemic pathological state triggered by exposure to elevated ambient temperatures or intense physical exertion. It is characterized by an imbalance between heat production and dissipation, which arises when endogenous heat production exceeds the body’s capacity for heat loss or when the thermoregulatory center becomes impaired [1,2]. The incidence of HS has risen dramatically over the past two decades, driven primarily by the persistent increase in global average temperatures associated with climate change. This trend poses a significant threat to both human health and economic livestock production [3,4,5,6]. During HS, blood flow is redistributed to enhance heat exchange between the skin and the external environment, resulting in substantial peripheral vasodilation and blood shunting [2,7]. However, this compensatory response often compromises perfusion of vital organs. As a result, HS frequently induces secondary injury in multiple organs, including the heart, liver, and kidneys [8,9,10,11]. At the molecular level, HS-induced organ injury is closely associated with oxidative excessive stress, activation of inflammatory cascades, and dysregulation of programmed cell death pathways [12,13,14]. These processes involve several key signaling pathways, such as the p38 mitogen-activated protein kinase (MAPK) signaling pathway, the nuclear factor-κB (NF-κB) signaling pathway, and the caspase cascade [15,16,17]. Collectively, these findings highlight the urgent need for effective therapeutic strategies to prevent or mitigate HS-induced organ injury. However, current clinical management of HS relies primarily on physical cooling and fluid resuscitation, which often fail to halt the progression of multi-organ dysfunction syndrome [8,18].

*Sophora flavescens* Ait. (Kushen), a traditional medicinal herb from the Fabaceae family, is widely recognized for its therapeutic potential in managing HS-related conditions [19]. Oxymatrine, the predominant quinolizidine alkaloid found in its dried roots, is considered a key bioactive constituent [20]. While crude extracts of *S. flavescens* have demonstrated efficacy in mitigating thermal injury in mammals, the precise active ingredients that drive these protective effects have not been definitively characterized. Literature suggests that oxymatrine exerts potent cytoprotective effects across various injury models. For instance, oxymatrine pretreatment improves renal outcomes and suppresses oxidative stress and apoptosis via the Nrf2/HO-1 pathway in ischemia–reperfusion models [21]. In acute liver failure, it mitigates hepatocyte apoptosis through the modulation of TLR4/PI3K/Akt/GSK-3β signaling [22]. Similar protective mechanisms involving the PI3K/Akt pathway have been observed in oxymatrine-treated H9c2 cardiomyocytes subjected to hypoxia/reoxygenation [23]. Emerging evidence also highlights oxymatrine’s capacity to regulate ferroptosis through the SIRT1/YY1/GPX4 axis, expanding its known regulatory repertoire [24]. Considering its multifaceted pharmacological profile—encompassing anti-inflammatory, antioxidant, and anti-apoptotic activities—and its demonstrated multi-organ protective capacity [25], we propose that oxymatrine serves as a major active component underlying the thermoprotective properties of *S. flavescens*. Moreover, the well-established industrial extraction of oxymatrine facilitates its potential clinical application and commercial development [26].

Systems pharmacology and computational ligand–protein interaction modeling have evolved into robust methodologies for deciphering pharmacodynamic mechanisms. The former discipline, rooted in integrative systems biology, specializes in dissecting intricate molecular interaction networks and highlighting hierarchical control of intracellular signaling routes. By integrating drug–target–disease interaction networks, it enables systematic prediction of potential targets and identification of key pathways involved in drug action [27,28]. Molecular docking is a computational technique widely used to predict the binding affinity and interaction patterns between small-molecule ligands and target proteins. It provides structural insights that facilitate the screening and validation of candidate therapeutic compounds [29].

**Figure 1:**
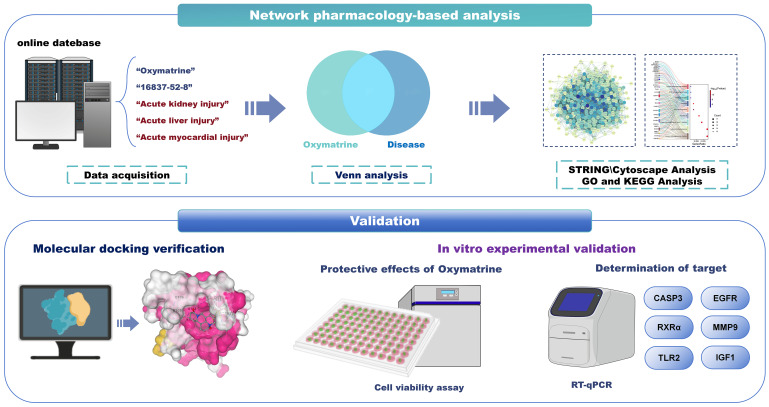
Flowchart of the study design.

In this study, we integrated network pharmacology analysis, molecular docking simulations, and in vitro cellular experiments to systematically elucidate the protective effects of oxymatrine against HS-induced kidney, liver, and myocardium. Furthermore, we explored its potential mechanisms, with a particular focus on the PI3K/Akt signaling pathway and ferroptosis regulation. The overall study design is illustrated in Figure 1.

## 2. Results

### 2.1. Identification of Core Targets in Heat Stress-Induced Organ Injury

**Figure 2:**
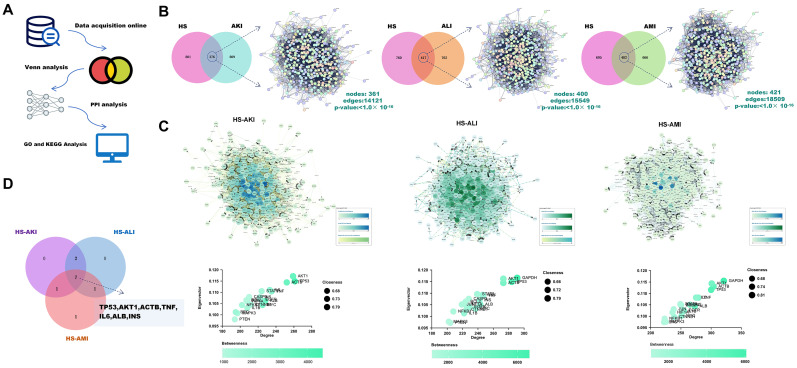
Identification of core targets in HS-induced organ injury. (**A**) Workflow for the acquisition and analysis of HS-induced organ injury targets. (**B**) Venn diagram analysis of common targets between “heat stress” and “acute kidney injury”, “acute liver injury”, and “acute myocardial injury”, respectively. (**C**) Analysis of disease core targets using four topological algorithms of the CytoNCA plugin in Cytoscape software (version 3.10.3). (**D**) Venn diagram analysis of core targets among the three diseases.

The workflow for identifying and analyzing heat stress-induced organ injury-related targets is illustrated in Figure 2A. The number of targets retrieved from each database based on keywords is presented in Table 1. For datasets with fewer than 1000 targets, all entries were included. After applying these criteria and removing duplicate entries, a total of 1177 targets were obtained for “heat stress”, 1185 for “acute kidney injury”, 1179 for “acute liver injury”, and 1148 for “acute myocardial injury”.

Venn diagram analysis identified 376 overlapping targets between “heat stress” and “acute kidney injury”, 417 between “heat stress” and “acute liver injury”, and 482 between “heat stress” and “acute myocardial injury” (Figure 2B). For each disease, the common targets were submitted to STRING (version 12.0; *Homo sapiens*; confidence threshold > 0.4) to build PPI networks (Figure 2C). These networks were then visualized in Cytoscape (version 3.10.3), and the CytoNCA plugin was used to compute four topological metrics (Betweenness, Closeness, Degree, Eigenvector) for core target identification (Figure 2C). The top 10 core targets for each of the three diseases were subjected to Venn analysis, ultimately yielding seven common core targets: TP53, AKT1, ACTB, TNF, IL6, ALB, and INS (Figure 2D).

### 2.2. Validation of Core Targets and Analysis of Pathogenic Pathways in Heat Stress-Induced Organ Injury

**Figure 3:**
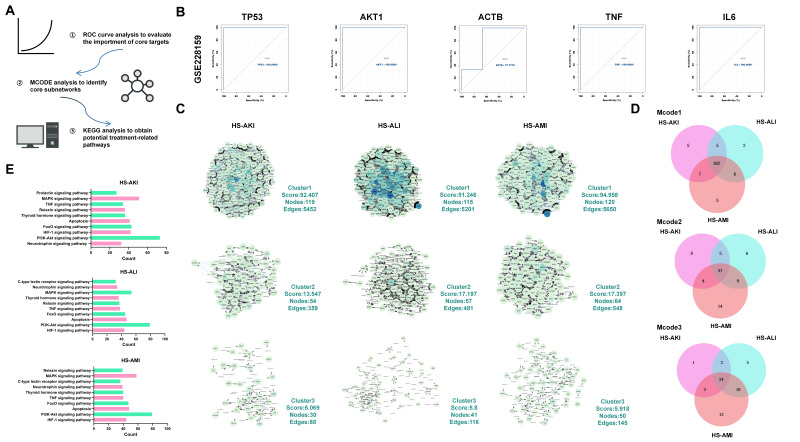
Validation of core targets and analysis of pathogenic pathways in HS-induced organ injury. (**A**) Workflow of core target validation and functional enrichment analysis. (**B**) ROC curve analysis results for TP53, AKT1, ACTB, IL6, TNF based on the GSE228159 dataset. (**C**) MCODE module clustering results of PPI networks in HS-AKI, HS-ALI, and HS-AMI, showing the top three clusters by score. (**D**) Intersection analysis of clustering modules across the three diseases. (**E**) Major pathogenic pathways identified by KEGG enrichment analysis.

The workflow for validating core targets and analyzing pathogenic pathways in HS-induced organ injury is illustrated in Figure 3A. Based on the GSE228159 dataset, receiver operating characteristic (ROC) curve analysis was performed to evaluate the diagnostic performance of the identified core targets. It should be noted that the GSE228159 dataset did not contain valid expression data for ALB and INS. The results demonstrated that TP53, AKT1, ACTB, IL6, and TNF all exhibited area under the curve (AUC) values greater than 0.7. This finding indicates that these genes have good discriminatory ability, as shown in Figure 3B. The MCODE plugin in Cytoscape software (version 3.10.3) was used to perform module clustering analysis on the three PPI networks. Three major functional modules were identified. These modules were designated as Cluster 1, Cluster 2, and Cluster 3. All three clusters were highly conserved across HS-AKI, HS-ALI, and HS-AMI. Among them, Cluster 1 was identified as the core functional module, while Clusters 2 and 3 were classified as secondary modules, as shown in Figure 3C. Intersection analysis of the three sets of modules further revealed a common core network underlying multi-organ injury (Figure 3D). KEGG pathway analysis used the DAVID database. The results showed that the main pathogenic pathways associated with HS-induced organ injury include the PI3K/AKT signaling pathway, the MAPK signaling pathway, and the apoptosis pathway, as shown in Figure 3E.

### 2.3. Oxymatrine Exhibits Potential Protective Effects Against Heat Stress-Induced Injury

**Figure 4:**
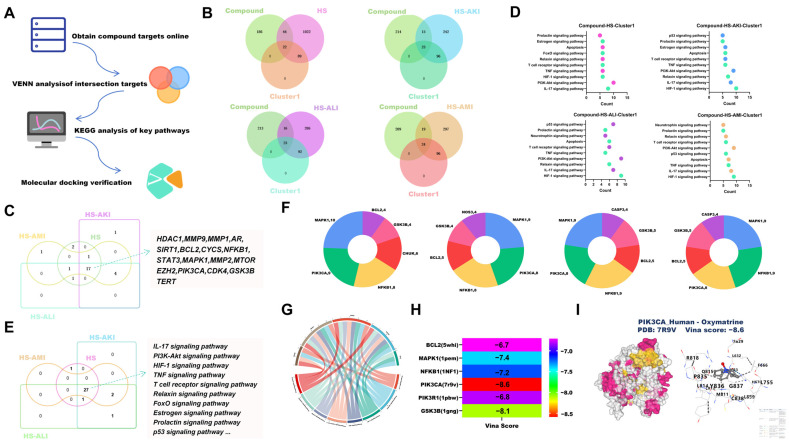
Systems pharmacology investigation of oxymatrine’s therapeutic potential against thermal injury. (**A**) Computational screening pipeline schematic. (**B**) overlap between alkaloid candidate targets and pathological target sets for generalized thermal stress, thermal stress-complicated renal failure, hepatic damage, cardiac injury, plus the central functional module; (**C**) quadruple intersection of overlapping target subsets; (**D**) functional pathway enrichment for each intersection set; (**E**) consensus pathway identification across four enrichment profiles. (**F**) Frequency distribution of recurrently appearing genes within top-ranked KEGG pathways. (**G**) connectivity plot illustrating target–pathway associations. (**H**) In silico binding affinity validation summary. (**I**) Three-dimensional structural representation of oxymatrine-PIK3CA complex formation.

To elucidate the mechanistic basis for oxymatrine’s protective action against thermal damage, a comprehensive bioinformatic investigation was conducted, with the procedural schematic presented in Figure 4A. A total of 252 targets were obtained using the keyword “oxymatrine”. These 252 targets were subjected to Venn analysis with the disease targets of HS, HS-AKI, HS-ALI, and HS-AMI, as well as with the targets of the core functional module (Cluster 1). This analysis identified 22, 23, 23, and 24 overlapping targets, respectively, as shown in Figure 4B. Subsequently, the intersection of the above four sets of overlapping targets was taken, ultimately identifying 17 common targets, suggesting that these are the core targets of oxymatrine against heat stressinjury (Figure 4C). KEGG pathway enrichment analysis was performed for each drug–disease intersecting target set. The results indicated that oxymatrine may exert protective effects against HS-induced injury through multiple signaling pathways. These pathways mainly include the PI3K-AKT signaling pathway, the HIF-1 signaling pathway, and the IL-17 signaling pathway, as shown in Figure 4D. Venn analysis of the four KEGG enrichment results revealed a substantial overlap among the associated pathogenic pathways (Figure 4E). The high-frequency genes among the top 10 pathways in each enrichment result were further identified. PIK3CA, BCL2, NFKB1, MAPK1, and GSK3B were recognized as key hub genes with high occurrence frequency (Figure 4F–G). Molecular docking analysis demonstrated that oxymatrine exhibited strong binding affinity with these core targets. All vina scores were lower than −6 kcal/mol, indicating stable interactions between oxymatrine and the target proteins, as shown in Figure 4H,I.

### 2.4. Oxymatrine Exhibits Potential Protective Effects on 293T Cells and H9C2 Cells Under Heat Stress

**Figure 5:**
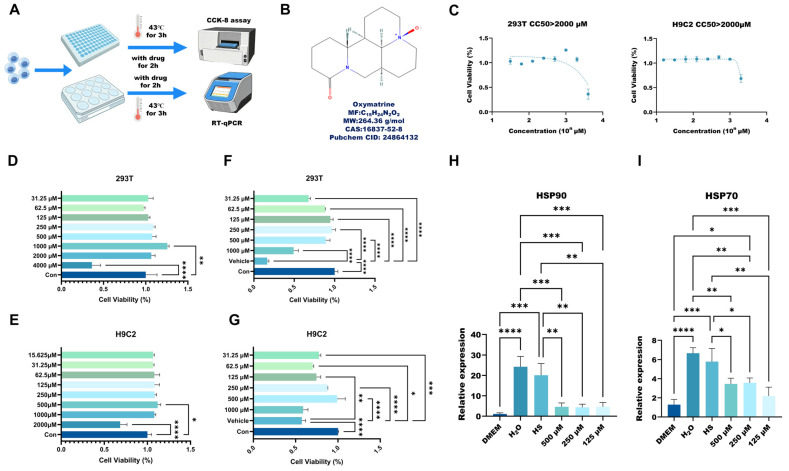
Cytoprotective efficacy of oxymatrine in Heat-stressed 293T cells and H9C2 cells. (**A**) Experimental procedure flowchart. (**B**) Chemical structure and molecular information of oxymatrine. (**C**) Dose–Response curves indicating the CC_50_ of oxymatrine in both cell lines. (**D**,**E**) Cell viability of 293T (**D**) and H9C2 (**E**) cells treated with indicated concentrations of oxymatrine for 48 h. (**F**,**G**) Quantitative assessment of prophylactic oxymatrine treatment effects on cell viability following heat stress, measured via the CCK-8 colorimetric assay in 293T (**F**) and H9C2 (**G**) cells. (**H**) Relative mRNA expression level of *HSP90*. (**I**) Relative mRNA expression level of *HSP70*. Significance thresholds: * *p* < 0.05, ** *p* < 0.01, *** *p* < 0.001, **** *p* < 0.0001.

The experimental workflow and the chemical structure of oxymatrine are illustrated in Figure 5A and Figure 5B, respectively. To evaluate the biocompatibility of oxymatrine, 293T cells were exposed to a concentration gradient ranging from 31.25 to 4000 μM, while H9C2 cells were exposed to concentrations from 15.625 to 2000 μM. CCK-8 assay results showed that the maximum safe concentrations for 293T and H9C2 cells were 1000 μM and 500 μM, respectively, with the half-maximal cytotoxic concentration (CC_50_) exceeding 2000 μM for both cell lines (Figure 5C–E). Based on these safety profiles, concentrations of 1000 μM and below were selected for subsequent experiments.

To assess the thermoprotective effects of oxymatrine, cells were subjected to heat stress at 43 °C for 3 h. CCK-8 results demonstrated that oxymatrine treatment at concentrations of 62.5–500 μM significantly enhanced cell viability compared to the vehicle group, restoring it to levels comparable to the control group (Figure 5F,G).

Furthermore, RT-qPCR analysis of key heat stress markers revealed that heat stress significantly upregulated the mRNA expression of *HSP90* and *HSP70* in 293T cells. In contrast, pre-treatment with oxymatrine (125–500 μM) markedly downregulated the expression of these markers, with no significant differences observed among the various oxymatrine-treated groups (Figure 5H,I).

### 2.5. The PI3K-AKT Signaling Pathway May Contribute to the Protective Action of Oxymatrine

**Figure 6:**
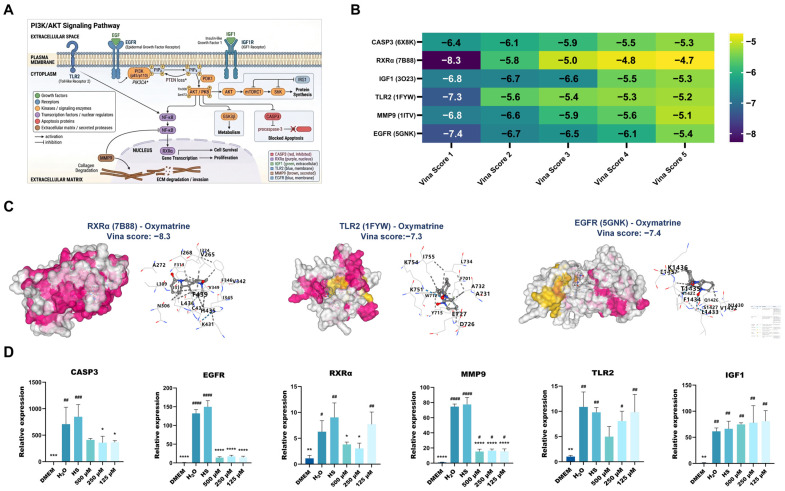
Oxymatrine modulates PI3K-AKT pathway gene expression and binds key proteins. (**A**) Schematic of PI3K-AKT signaling. (**B**) Docking scores for oxymatrine with pathway proteins. (**C**) Binding poses with *RXRα*, *TLR2*, and *EGFR*. (**D**) mRNA levels of *CASP3*, *EGFR*, *RXRα*, *MMP9*, *TLR2*, and *IGF1*. Statistical significance was denoted as * *p* < 0.05, ** *p* < 0.01, *** *p* < 0.001, **** *p* < 0.0001; # *p* < 0.05, ## *p* < 0.01, ### *p* < 0.001, #### *p* < 0.0001, * indicating comparison to DMEM-treated cells, # referring to comparison against HS group.

Based on the preceding network pharmacology findings, the PI3K-AKT signaling cascade was predicted to be involved in oxymatrine’s protective effect against heat stress in 293T cells (Figure 6A provides a schematic overview of this pathway). To test this hypothesis, we performed molecular docking between oxymatrine and several key components of this pathway, namely *CASP3*, *EGFR*, *RXRα*, *MMP9*, *IGF1*, and *TLR2*. oxymatrine displayed strong binding affinity to all tested targets (Figure 6B,C). Next, reverse transcription quantitative PCR (RT-qPCR) was used to measure the mRNA levels of these genes. Exposure to heat stress markedly upregulated the transcript levels of *CASP3*, *EGFR*, *RXRα*, and *MMP9*. Treatment with oxymatrine dose-dependently reversed these elevations (Figure 6D). In contrast, *TLR2* expression tended to decline as oxymatrine concentration increased, although this trend did not reach statistical significance. Meanwhile, oxymatrine had no notable effect on the heat-induced upregulation of *IGF1* expression.

### 2.6. Potential Mechanism of Oxymatrine in Regulating Ferroptosis

**Figure 7:**
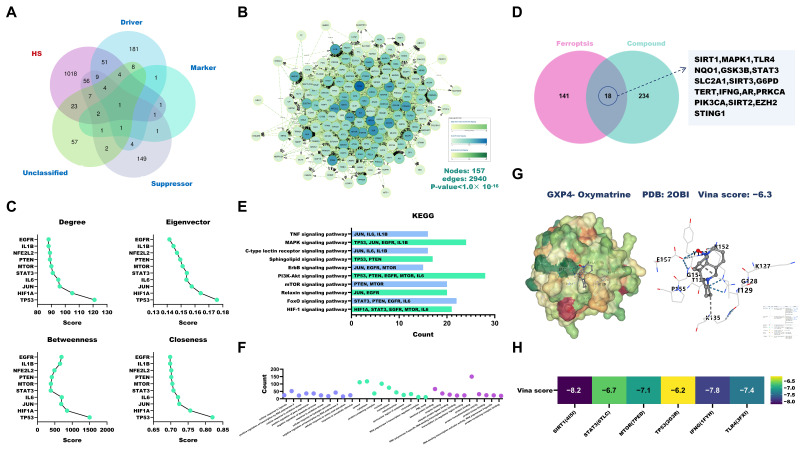
Oxymatrine regulates ferroptosis via network pharmacology. (**A**) HS and ferroptosis target overlap. (**B**) PPI network of shared targets. (**C**) CytoNCA core target analysis. (**D**) Oxymatrine–ferroptosis target intersection. (**E**,**F**) KEGG and GO enrichment. (**G**) Oxymatrine-GPX4 docking details. (**H**) Validation of ferroptosis-related binding.

As shown in Figure 7A, a Venn diagram analysis uncovered 159 common targets shared between HS and ferroptosis-associated genes. A protein–protein interaction (PPI) network was then built using the STRING database and subsequently loaded into Cytoscape (version 3.10.3). Core target analysis was performed with the CytoNCA plugin, leading to the identification of the top 10 key regulators (Figure 7B,C). Venn analysis showed that oxymatrine shared 18 targets with ferroptosis, and notably, eight of these targets overlapped with the core targets of oxymatrine against heat stress injury identified earlier (Figure 7D). KEGG pathway enrichment analysis and GO functional annotation revealed substantial overlap between the signaling pathways enriched by the ferroptosis-related intersecting targets and those enriched by the core targets of oxymatrine against heat stress injury. This preliminary bioinformatic association suggests that oxymatrine might tentatively exert its protective effects through a multi-pathway framework that includes ferroptosis-related signaling. (Figure 7E,F). Molecular docking analysis showed that oxymatrine had a Relatively strong binding affinity for GPX4 (Vina score: −6.3 kcal/mol) (Figure 7G). Oxymatrine also exhibited high binding affinities for key targets such as SIRT1, STAT3, MTOR, TP53, IFNG, and MAPK1 (Vina scores < −6.5 kcal/mol) (Figure 7H).

## 3. Discussion

In the present work, a combined strategy of network pharmacology, molecular docking, and cellular assays was employed to assess the cytoprotective potential of oxymatrine under heat stress (HS). Our findings indicate that oxymatrine mitigates HS-inflicted injury via the coordinated engagement of multi-pathway mechanisms. Particularly noteworthy is the convergent modulation of the PI3K-AKT survival axis and iron-dependent cell death processes, which appears to constitute a fundamental mechanistic basis for the observed cytoprotection. In this study, targets associated with HS-induced AKI, ALI, and AMI were obtained from multiple databases. A PPI network was subsequently constructed. Topological analysis using four algorithms identified key hub targets, including GAPDH, ACTB, AKT1, TP53, TNF, ALB, IL6, INS, IL1B, and EGFR. Venn analysis further revealed that seven core genes (TP53, AKT1, ACTB, TNF, IL6, ALB, and INS) were shared across all three disease types. This suggests that HS-induced multi-organ injury may be driven by conserved molecular mechanisms. Moreover, ROC curve analysis validated the diagnostic potential of the identified targets. Due to the absence of expression data for ALB and INS in the GSE228159 dataset, these two genes could not be validated. However, for the remaining five key genes, the AUC values exceeded 0.7, indicating reliable diagnostic performance. MCODE clustering revealed three highly conserved functional modules shared among different organ injuries, implying the existence of a unified pathological network. Moreover, KEGG enrichment analysis identified the PI3K-AKT signaling pathway as a central mediator of heat stress pathology, corroborating previous studies [30,31].

Numerous studies have shown that heat stress triggers excessive generation of reactive oxygen species (ROS), leading to the activation of inflammatory cascades including the NLRP3/IL-1β/IL-6 axis [13,32]. Pro-inflammatory cytokines like TNF-α and IL-6 are known to briefly activate the PI3K-AKT pathway as an adaptive stress response [33,34,35]. However, when this activation becomes persistent, it can promote apoptosis and worsen tissue damage [36,37,38]. Therefore, therapeutically restraining aberrant PI3K-AKT signaling may be beneficial in HS-related injury. Our Venn analysis uncovered 17 overlapping targets between oxymatrine and HS-associated genes. KEGG enrichment pointed mainly to the PI3K-AKT, HIF-1, and IL-17 pathways. Molecular docking results demonstrated that oxymatrine interacts with high affinity with PIK3CA and GSK3B, yielding calculated binding energies of −8.5 and −8.1 kcal/mol, respectively. These findings lend structural credibility to the network pharmacology predictions.

To validate these findings, a heat stress cell model was established using 293T cells. Cytotoxicity assays showed that the half-maximal cytotoxic concentration (CC_50_) of oxymatrine in 293T cells exceeded 2000 μM, and 1000 μM was determined as the maximum safe concentration. Further experiments revealed that oxymatrine at concentrations ranging from 31.25 to 1000 μM exerted protective effects on heat-stressed 293T cells, with the most pronounced increase in cell viability observed in the concentration range of 62.5–500 μM, averaging approximately 30%. RT-qPCR results confirmed that oxymatrine significantly reversed the HS-induced upregulation of *HSP90* and *HSP70* mRNA expression.

Based on the KEGG enrichment analysis results and the previous studies, CASP3, EGFR, RXRα, MMP9, TLR2, and IGF1 were identified as key regulatory factors of the PI3K-AKT signaling pathway [39,40,41,42]. It is worth noting that while topological analysis identified hubs such as TP53, AKT1, these targets often capture highly nonspecific housekeeping or general inflammatory responses common to various pathological states. To ensure biological specificity, we prioritized the validation of these six genes, as they serve as specific upstream receptors or downstream functional executors that more accurately reflect the specific pathological progression of HS-induced multi-organ injury and the targeted regulatory effects of oxymatrine. The RT-qPCR results of this study showed that oxymatrine effectively reversed the HS-induced upregulation of *CASP3*, *EGFR*, *RXRα*, and *MMP9* mRNA expression, but had no significant effect on *IGF1* expression. As an upstream receptor tyrosine kinase of the PI3K-AKT signaling pathway, EGFR can interact with the IGF signaling pathway, leading to dimerization and autophosphorylation [43]. This process regulates PI3K and AKT signaling molecules through downstream proteins, subsequently modulating the activity of the nuclear receptor RXRα and promoting the expression of the anti-apoptotic gene BCL-2 [44,45]. Consequently, the activation of caspase-3 is inhibited, thereby reducing apoptosis [46]. In addition, TLR2 can also inhibit apoptosis by activating this pathway [47]. On the other hand, activation of EGFR and TLR2 can directly or indirectly stimulate MMP9 production through activation of the NF-κB signaling pathway, thereby promoting apoptosis [48,49,50]. Although docking predicted strong binding to IGF1 and TLR2, RT-qPCR showed no significant effect on IGF1 expression and a non-significant trend for TLR2. This may reflect limitations of docking or cell-type specific regulation. Future studies should examine protein-level changes and alternative downstream effectors. Therefore, oxymatrine can protect heat-treated 293T cells from apoptosis by inhibiting the expression of EGFR- and RXRα-related synthetic genes. However, Protein-level validation was not performed; therefore, the proposed pathway modulation remains at the transcriptional level and should be considered preliminary.

Although ferroptosis was not identified among the top enriched KEGG pathways, several core targets, such as TP53 and AKT1, have been reported to modulate ferroptosis. Coupled with molecular docking showing strong binding between oxymatrine and GPX4, we proceeded to investigate whether oxymatrine alleviates heat stress injury partly through ferroptosis inhibition. A total of 159 overlapping targets were identified between HS-related genes and ferroptosis-related genes. Oxymatrine shared 18 common targets with the ferroptosis pathway, eight of which overlapped with the aforementioned core targets against heat stress injury. Molecular docking results showed that oxymatrine exhibited strong binding affinity for GPX4, a key regulatory enzyme in ferroptosis (Vina score: −6.3 kcal/mol), and also bound well to ferroptosis regulators such as SIRT1, STAT3, and TP53. These findings suggest that oxymatrine may exert protective effects against HS-induced multi-organ injury through coordinated regulation of the PI3K-AKT signaling pathway and the ferroptosis process. It is noteworthy that previous studies reported oxymatrine triggers ferroptosis in liver cancer cells through the SIRT1/YY1/GPX4 axis [24]. This contrasts with our findings but highlights the context-dependent nature of oxymatrine. In the anti-tumor context, oxymatrine exerts pro-oxidant and pro-apoptotic effects; however, in the context of organ protection under exogenous heat stress, oxymatrine acts as a homeostatic regulator to maintain cell survival and inhibit pathological cell death pathways.

Despite the evidence pointing to oxymatrine’s therapeutic promise against HS-induced injury, certain limitations must be considered. First, the in vitro experiments were mainly based on the 293T and H9C2 cell lines, and the protective effects of oxymatrine against heat stress need to be validated in animal models to confirm their clinical relevance. Second, further investigation of the precise molecular interactions within the PI3K-AKT pathway will contribute to a more comprehensive understanding of how oxymatrine modulates inflammation and apoptosis. Third, functional validation at the protein level remains lacking; in particular, the anti-apoptotic effects are currently supported by RT-qPCR data for *CASP3* and network pharmacology predictions, which provide a useful preliminary framework but require further confirmation through protein-level assays and functional studies. In addition, the conclusions regarding ferroptosis are primarily derived from network pharmacology predictions and molecular docking simulations, and subsequent experimental validation through functional assays such as detection of lipid peroxidation product levels and GPX4 enzyme activity is required. Despite these limitations, the favorable safety profile of oxymatrine in vitro and its regulatory effects on stress-related gene expression suggest promising clinical potential. In particular, these findings suggest that oxymatrine is a promising candidate for further evaluation in HS-related protection, pending in vivo validation.

## 4. Materials and Methods

### 4.1. Cells and Compound

The 293T cell line and H9C2 cell line were acquired from the China Center for Type Culture Collection (CCTCC, Wuhan, China). Oxymatrine (purity > 99%) was purchased from MedChemExpress (Monmouth Junction, South Brunswick, NJ, USA; Cat. No. HY-N0158).

### 4.2. Network Pharmacology Analysis

#### 4.2.1. Acquisition of Disease Targets and Drug Targets

We retrieved disease-related targets by searching four public databases—GeneCards, OMIM, MalaCards, and STRING—using the keywords “heat stress”, “acute kidney injury”, “acute liver injury”, and “acute myocardial injury” individually, limiting the search to Homo sapiens. For each query, if the number of hits exceeded 1000, only the top 1000 were taken; otherwise, all hits were included. After merging and deduplicating the data from the four sources, we obtained four independent target sets corresponding to HS, AKI, ALI, and AMI [51,52,53,54].

Potential targets of oxymatrine were predicted using the following databases: Traditional Chinese Medicine Systems Pharmacology Database and Analysis Platform (TCMSP), SuperPred, HERB, TargetNET, and SwissTargetPrediction Data from each source were pooled and duplicate entries were discarded [55,56,57,58,59].

#### 4.2.2. Construction of PPI Network and Identification of Core Targets

Overlaps between oxymatrine targets and each disease target set were calculated with the jVenn online tool [60]. The intersecting targets were then submitted to STRING (setting: Homo sapiens, confidence score ≥ 0.4) to build protein–protein interaction (PPI) networks, and free nodes without interactions were hidden. The resulting PPI networks were visualized in Cytoscape (version 3.10.3), where node size and fill color were scaled according to degree centrality [61,62].

Within Cytoscape 3.10.3, we employed the CytoNCA plugin to compute four node-level centrality parameters: betweenness, closeness, degree, and eigenvector. A consensus of these four algorithms was then used to define the core targets. Module clustering analysis was performed using the MCODE plugin with default parameters and a module score threshold of ≥2, and the top three clustering modules by score were retained for visualization.

#### 4.2.3. KEGG Enrichment Analyses

KEGG pathway enrichment was carried out using the DAVID database, with the species background set to *Homo sapiens*. The top 10 terms (by ascending *p*-value) for KEGG were selected. Visualization employed the Wei Sheng Xing platform [63,64].

#### 4.2.4. Molecular Docking Simulation

We retrieved crystal structures of target protein from the RCSB PDB database, filtering for *Homo sapiens*, the experimental method limited to X-ray crystallography, and a resolution screening criterion of <3.0 Å. The structure of oxymatrine was downloaded from the PubChem database. Blind docking simulations employed CB-DOCK2 online platform with default cavity detection. Binding energies were calculated as Vina scores (kcal/mol); optimal poses were selected for structural visualization. CB-Dock2 performs dual blind docking and selects the optimal pose based on Vina score and RMSD (<2 Å) when a template is available. It should be noted that Vina scores are for predictive purposes only and do not represent experimentally validated binding affinities [61,65,66,67].

### 4.3. In Vitro Cellular Experiments

#### 4.3.1. Culture Conditions

293T cells and H9C2 cells were maintained in Dulbecco’s Modified Eagle Medium (DMEM; Beijing Solarbio Science & Technology Co., Ltd., Beijing, China) supplemented with 10% FBS, 100 U/mL penicillin G, and 100 μg/mL streptomycin sulfate. Incubation conditions comprised 37 °C, 5% CO_2_, and saturated humidity. Passages were conducted at 48–72 h intervals.

#### 4.3.2. Cytotoxicity Assay

For cytotoxicity testing, 293T cells and H9C2 cells were seeded into 96-well plates at 1 × 10^4^ cells/well in 200 μL of medium. After 24 h of attachment, the medium was replaced with fresh medium containing serial dilutions of oxymatrine (for 293T: 31.25, 62.5, 125, 250, 500, 1000, 2000, and 4000 μM; for H9C2: 15.625, 31.25, 62.5, 125, 250, 500, 1000, and 2000 μM). A vehicle control (DMSO, final concentration < 0.1%) was included, and each concentration was tested in six replicate wells.

Following 48 h of treatment, 10 μL of CCK-8 reagent (purchased from Vazyme Biotech Co., Ltd., Nanjing, China) was added per well, and the plates were kept in darkness at 37 °C for 2–2.5 h. A microplate reader was used to measure the absorbance at 450 nm, from which the percentage of viable cells was computed. Dose–Response curves were fitted using GraphPad Prism 10.6.0 to determine the half-maximal cytotoxic concentration (CC_50_). The entire procedure was repeated three independent times.

#### 4.3.3. Establishment of Heat Stressed Cell Model and Drug Treatment

For 293T cells, the experiment comprised eight groups: a normal control group (37 °C, no treatment), a vehicle group (DMEM, followed by heat stress), and six oxymatrine-treated groups (31.25, 62.5, 125, 250, 500, and 1000 μM, each followed by heat stress). Each group included six replicate wells. 293T cells were plated at 1 × 10^4^ cells/well in 96-well plates and allowed to attach for 24 h. The culture medium was then removed and replaced with fresh medium containing the indicated concentrations of oxymatrine or an equivalent volume of vehicle, according to the experimental design. Subsequently, cells in the heat-stress groups were shifted to 43 °C in a humidified 5% CO_2_ incubator for 3 h, while control cells remained at 37 °C. After the thermal challenge, the medium was replaced with serum-free DMEM. CCK-8 solution (10 μL per well) was added, and plates were incubated for another 2–2.5 h. Absorbance at 450 nm was recorded, and cell viability was calculated.

For H9C2 cells, the same experimental design was applied, with the following modifications: the tested oxymatrine concentrations were 15.625, 31.25, 62.5, 125, 250, and 500 μM. All other procedures were identical to those described for 293T cells.

#### 4.3.4. Quantitative Real-Time PCR (RT-qPCR)

Following the specified treatments, the culture supernatant was discarded, and the cells were rinsed three times with PBS before being collected by centrifugation. Total RNA was isolated with the FastPure Cell/Tissue Total RNA Isolation Kit V2 according to the manufacturer’s instructions. RNA purity was evaluated by measuring the OD_260_/OD_280_ ratio, with values between 1.8 and 2.0 considered acceptable. A total of 1 μg of RNA was then reverse-transcribed into cDNA using ExonScript RT SuperMix containing dsDNase (Beijing Yijiaren Biotechnology Co., Ltd., Chengdu, China).

qPCR was performed with TB Green^®^ Premix Ex Taq^TM^ II FAST qPCR reagents (Takara Bio Inc., Dalian, China) according to the manufacturer’s instructions. Three independent biological replicates were performed, each with triplicate technical repeats. Cycle threshold (Ct) values ranged from 28 to 35 across all samples, and no-template controls yielded no detectable amplification. Transcript quantification was normalized to ACTIN endogenous control. Fold-change calculations employed the comparative 2^(−ΔΔCt)^ algorithm. Oligonucleotide primers (Supplementary Table S1) were custom-synthesized by Sangon Biotech (Shanghai, China).

### 4.4. Statistical Analysis

Statistical inference relied on single-factor analysis of variance computed via GraphPad Prism software (version 10.6.0). Probability values below 0.05 were deemed statistically significant.

## 5. Conclusions

Collectively, the convergence of computational predictive modeling and empirical verification utilizing a thermally challenged human embryonic kidney cell system demonstrates that oxymatrine confers protection against hyperthermic insult primarily through modulation of the PI3K-AKT signaling network, with additional evidence suggesting involvement of ferroptotic pathway regulation.

## Figures and Tables

**Table 1:** Summary of target numbers retrieved from each database for different disease keywords.

Database	Heat Stress	Acute Kidney Injury	Acute Liver Injury	Acute Myocardial Injury
GeneCards	11,200 (Top 1000)	12,491 (Top 1000)	12,461 (Top 1000)	5914 (Top 1000)
OMIM	274 (all)	249 (all)	250 (all)	313 (all)
MalaCards	2 (all)	37 (all)	8 (all)	59 (all)
String	89 (all)	24 (all)	3 (all)	8 (all)
Total after deduplication	1177	1185	1179	1148

## Data Availability

The original contributions presented in this study are included in the article/Supplementary Materials. Further inquiries can be directed to the corresponding authors.

## Supplementary Materials

The following supporting information can be downloaded at: https://www.mdpi.com/article/10.3390/ijms27135919/s1.
