# Supplementary material for: Investigating the Potential Mechanism of Oxymatrine in Alleviating Heat Stress Injury Based on Network Pharmacology, Molecular Docking, and In Vitro Validation
Source: Int J Mol Sci. 2026 Jun 30;27(13):5919. doi: 10.3390/ijms27135919 (PMC13362203; doi:10.3390/ijms27135919)
Supplement: Supplementary file 1 [file ijms-27-05919-s001.zip › Supplementary Material S3.pdf]

Certificate of Analysis

Oxymatrine

Cat. No.: HY-N0158

CAS No.: 16837-52-8

Batch No: 983653

Product name: 1H,5H,10H-Dipyrido[2,1-f:3',2',1'-ij][1,6]naphthyridin-10-one, dodecahydro-, 4-oxide, (4R,7aS,13aR,13bR,13cS)-

PHYSICAL AND CHEMICAL PROPERTIES

Formula: $C_{15}H_{24}N_2O_2$

Molecular Weight: **264.37**

Storage Conditions:

Powder	-20°C	3 years
	4°C	2 years
Insolvent	-80°C	6 months
	-20°C	1 month

Chemical Structure:

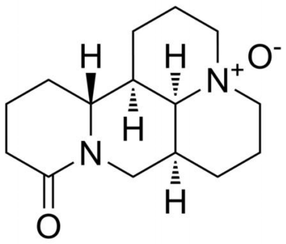

ANALYTICAL DATA

Appearance: White to off-white (Solid)

^{1}H NMR Spectrum: Consistent with structure

MS: Consistent with structure

Purity (HPLC): **99.92%**

Conclusion: The product has been tested and complies with the given specifications.

Caution: Product has not been fully validated for medical applications. For research use only.

Tel: 609-228-6898 Fax: 609-228-5909 E-mail: tech@MedChemExpress.com

Address: 1 Deer Park Dr, Suite F, Monmouth Junction, NJ 08852, USA

Inhibitors • Screening Libraries • Proteins
