# Supplementary material for: Investigating the Potential Mechanism of Oxymatrine in Alleviating Heat Stress Injury Based on Network Pharmacology, Molecular Docking, and In Vitro Validation
Source: Int J Mol Sci. 2026 Jun 30;27(13):5919. doi: 10.3390/ijms27135919 (PMC13362203; doi:10.3390/ijms27135919)
Supplement: Supplementary file 1 [file ijms-27-05919-s001.zip › Supplementary Table S1.pdf]

CASP3
CATGGAAGCGAATCAATGGACT
CTGTACCAGACCGAGATGTCA

EGFR
AGGCACGAGTAACAAGCTCAC
ATGAGGACATAACCAGCCACC

RXRA
GACGGAGCTTGTGTCCAAGAT
AGTCAGGGTTAAAGAGGACGAT

MMP9
TGTACCGCTATGGTTACACTCG
GGCAGGGACAGTTGCTTCT

TLR2
ATCCTCCAATCAGGCTTCTCT
GGACAGGTCAAGGCTTTTTACA

IGF1
GCTCTTCAGTTCGTGTGTGGA
GCCTCCTTAGATCACAGCTCC
